# Research Status of Metal–Organic Frameworks in Field of Membrane Distillation

**DOI:** 10.3390/membranes16080255

**Published:** 2026-07-27

**Authors:** Shuhua Ma, Quanxing Liao, Shiai Xu, Guanglan Che, Haoyi Chen, Juan Li

**Affiliations:** 1 School of Environmental and Ecological Engineering, Qinghai Vocational Technical University, Xining 810016, China; 13099099303@163.com (Q.L.);; 2 National Collaborative Innovation Center for Salt Lake Resource Chemistry and Process Engineering, School of Chemical Engineering, Qinghai University, Xining 810016, China; saxu@ecust.edu.cn; 3 Qinghai Yanhu Indurstry Co., Ltd., Xining 810016, China

**Keywords:** membrane distillation, metal–organic framework membranes, membrane separation, desalination, current research status

## Abstract

Membrane distillation (MD) technology has become an effective solution to freshwater scarcity due to its low energy consumption, high separation efficiency, and ability to handle highly concentrated saline wastewater. Nevertheless, issues such as membrane wetting, membrane fouling, and low membrane flux severely limit its large-scale application. Composite membranes prepared using metal–organic framework (MOF) materials as fillers have become a research hotspot due to their advantages, such as permeable microporous channels, customizable pore structures, and modifiable active sites. These properties enable them to effectively reduce temperature polarization and concentration polarization phenomena. This article describes the characteristics of MOF materials and their current applications in the field of MD, with a comparative analysis of the applicability of MOF polycrystalline membranes and MOF composite membranes in MD, and discusses the working principle of MOFs in enhancing the performance of MD. Finally, the problems and challenges associated with the use of MOFs in MD applications are analyzed. This study aims to provide theoretical guidance for the application of MOF materials in the field of MD seawater desalination.

## 1. Introduction

**Figure 1:**
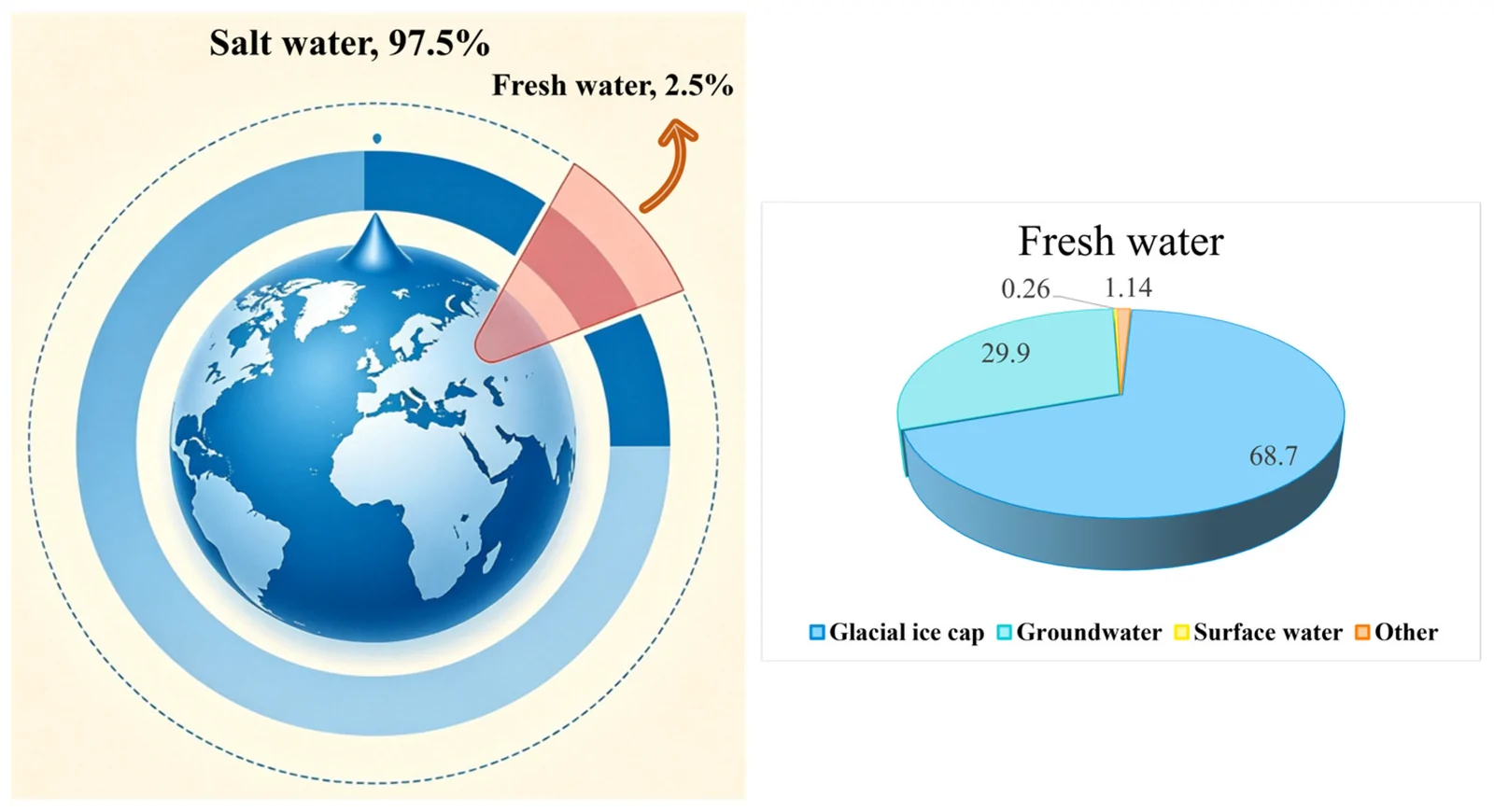
Distribution of water in Earth’s hydrosphere. Data form Chamani H., et al. 2021 [1].

Water resources, as a fundamental resource for life, are facing an unprecedented imbalance between supply and demand. As shown in Figure 1, the freshwater available for human use on Earth accounts for only 0.26% of the total water volume, and its distribution is extremely uneven [1]. The report “Global Water Bankruptcy: Survival Beyond Hydrological Carrying Capacity in the Post-Crisis Era” points out that about 2.2 billion people still lack access to safe drinking water, 3.5 billion people lack safely managed sanitation facilities, and nearly 4 billion people face severe water scarcity for at least one month each year. Globally, about three-quarters of the population live in countries where water resources are either insecure or extremely insecure. In this context, wastewater reuse [2] and desalination technology [3] serve as stable, economical, and sustainable solutions that provide important means to alleviate the global water crisis. Desalination refers to the process of removing dissolved minerals and salts from seawater to produce drinking water [4]. Since desalination does not depend on river flow, reservoir levels, or climate change, and since seawater accounts for 97% of the world’s total water resources, this technology has become a key approach in the global water market to meet freshwater demands [5]. Although reverse osmosis (RO) desalination technology has been industrialized, it still faces practical challenges, including high energy consumption and the generation of large volumes of highly saline wastewater. Therefore, there is an urgent need to find an economical and environmentally friendly alternative to address the global water shortage problem.

**Figure 2:**
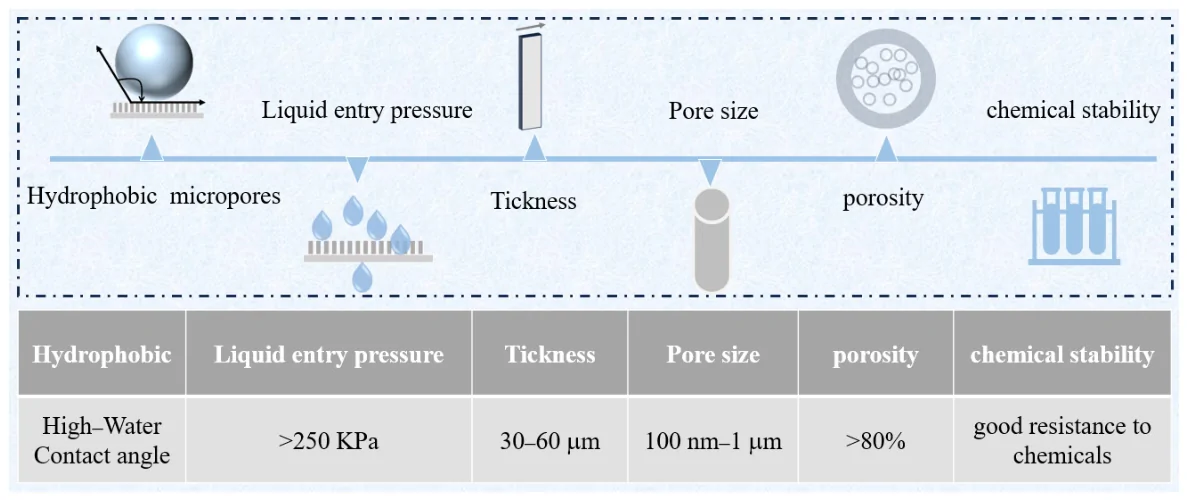
Summary of required characteristics for MD membrane.

Traditional seawater desalination methods are mainly divided into membrane-based and thermal processes [6]. Compared with thermal distillation processes, membrane processes have gained widespread attention for their advantages, including lower energy consumption, more compact equipment, and greater modularity [7]. The most common membrane technologies include forward osmosis (FO), RO, and MD [8]. Compared to RO technology, MD provides several advantages, including a higher salt rejection rate, lower operating pressures, and the ability to treat high-salinity brines that are challenging for RO systems. MD is a new separation technique that utilizes the vapor pressure difference across the membrane to drive the directional migration of water molecules. This technology combines membrane separation with evaporation processes and is being developed for industrial use. It specifically targets the global shortage of freshwater resources by offering an innovative solution. The core of membrane distillation is the development of specialized membrane materials with micron-sized pores and hydrophobic surfaces, effectively preventing liquid penetration while allowing water vapor to pass through. However, the MD membrane exhibits progressive wetting and low permeation flux, which significantly limit its practical applications [9], and membrane wetting reduces its salt rejection capability. When the membrane pores are wetted, the gas inside the pores is replaced by liquid. As a result, liquid on the feed-water side can pass freely through the pores into the permeate side, thereby reducing the desalination efficiency [10]. To achieve good wetting resistance, the MD membrane must have high hydrophobicity, small pore sizes, and no macroscopic pores to prevent water penetration. At the same time, to increase water flux, MD membranes need to have high porosity, low tortuosity, and an extremely thin thickness. Figure 2 lists the characteristic parameters of the MD membrane. It is necessary that it possesses key properties, such as low surface energy, high hydrophobicity [11], high liquid entry pressure [12], high porosity, an optimal membrane thickness [13], and an appropriate pore size. These properties are crucial for enhancing steam penetration and salt rejection.

Research shows that membrane materials affect membrane structures, which, in turn, influence membrane properties. Therefore, selecting the appropriate material for preparing MD membranes is crucial. Polymers are currently the preferred membrane material due to their low raw material and production costs, ease of processing, and suitability for mass production [14,15,16]. In recent years, due to the inherent “trade-off” characteristics of polymer membranes, the preparation and development of hybrid matrix membranes by dispersing nanoparticles as fillers within a polymer matrix have become popular among researchers. Table 1 summarizes the different types of membranes used in membrane distillation.

Since the discovery of graphene in 2004, two-dimensional (2D) materials have sparked great interest in numerous scientific fields [24]. Due to the high aspect ratio of two-dimensional materials, which results from their atomic thickness and micron-scale lateral dimensions, these materials are attracting increasing attention as a foundation for the production of high-performance membranes [25]. Among the existing nanomaterials, two-dimensional nanosheets are particularly suitable for use in membrane separation applications: their atomic-level thickness minimizes additional mass transfer resistance, while their larger lateral dimensions allow for effective control over the path of material transport [26,27]. Compared to traditional fillers, two-dimensional nanomaterials can provide extended and highly anisotropic interfaces, forming well-structured nanochannels that significantly enhance water permeability and solute retention capabilities [28,29]. However, it is technically challenging to create such a film without introducing defects into the polymer material.

First, the extremely thin membrane structure allows nanosheet membranes to exhibit a higher permeation rate than layered membranes. However, in practical applications, porous nanosheet membranes face greater challenges in terms of their membrane-sealing performance and durability. Second, the preparation of layered membranes is relatively easier. However, to achieve precise molecular separation (e.g., removing ions from water), significant challenges related to the control of transport channels still need to be overcome. Third, the stability of two-dimensional membrane materials and their scalable production processes must also be given due attention in order to advance their practical application [29]. From a surface engineering perspective, the abundant surface-active sites in MOFs enable them to exhibit multiple interfacial functions and allow for flexible modification strategies. This enables selective binding along with the creation of hydrophobic barriers, thereby suppressing non-specific interactions and membrane wetting. Two-dimensional nanomaterials have a single type of interface, resulting in limited control capabilities. Functionally, MOFs dominate separation and screening performance, while two-dimensional nanomaterials focus more on structure and mass transfer optimization. In comparison, MOFs feature organic–inorganic hybrid structures and customizable ligands, features such as precise control of pore size, efficient molecular separation, and diverse interfacial interactions, making its an ideal functional filler for high-performance separation membranes.

## 2. Metal–Organic Framework (MOF) Materials

### 2.1. Introduction to Metal–Organic Frameworks

**Figure 3:**
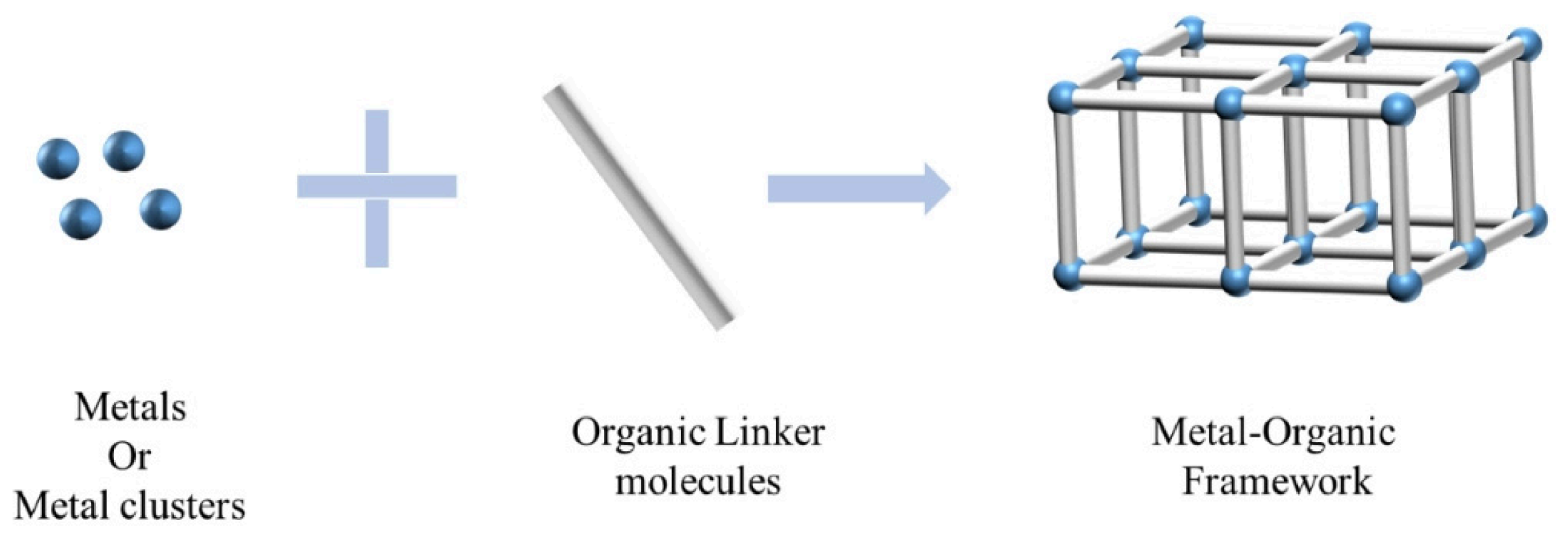
Schematic diagram of MOF structure.

MOFs are porous crystalline materials formed by organic ligands connecting inorganic building units, as shown in Figure 3. In 1995, Omar M. Yaghi published a study in the journal *Nature* describing a coordination compound with a two-dimensional structure, synthesized from the rigid organic ligand benzene-1,3,5-tricarboxylic acid (BTC) and the transition metal cobalt. He named this compound a metal–organic framework [30]. In 1999, the Yaghi research team reported in *Nature* a three-dimensional metal–organic framework material with a simple cubic structure MOF-5—which was constructed using rigid organic ligands to bind terephthalic acid (BDC) with the transition metal Zn. The emergence of MOF-5 material represents a milestone in the history of MOF development [31]. The modular design of porous materials was achieved (specific surface area of 2900 m^2^·g), laying the structural foundation for the development of MOFs [31,32,33].

### 2.2. Characteristics of Metal–Organic Frameworks

MOFs exhibit three significant advantages in the field of membrane distillation for seawater desalination. Firstly, the excellent porosity of MOFs (94%) gives them a unique advantage in the field of separation. Secondly, the structural units of MOFs combine with various metal ions/clusters through coordination bonds in different coordination modes, thereby endowing them with high structural diversity. Thirdly, the organic ligands in MOFs typically possess long-chain structures and σ-bond characteristics, and the vast majority of MOF materials exhibit significant flexibility [34]. In summary, MOFs’ advantages, such as their extremely high specific surface area, adjustable pore structure, controllable chemical properties, and excellent structural stability, have made them a revolutionary class of materials in the fields of materials science and chemistry. They hold great potential for applications across various disciplines [35,36,37,38,39]. Unfortunately, the powder-like nature of MOFs limits their application in the field of water treatment. First, MOF powders not only tend to aggregate but can also cause blockages that lead to secondary pollution. Second, powdered MOFs are prone to loss during practical applications, which not only shortens their lifespan but also increases operating costs. Third, immature production methods, unstable production cycles, and poor biocompatibility limit the widespread application of MOFs. Therefore, combining MOFs with regular pore structures with polymers that possess processability to create MMMs is highly favored by researchers.

### 2.3. MOF Mixed Matrix Membranes

The main reasons why mixed matrix membranes (MMMs) have a superior separation performance compared to pure polymers are as follows: Firstly, the addition of inorganic nanoparticles can reduce the entanglement of polymer molecular chains, decrease molecular chain aggregation, increase the free volume of the membrane, and thereby enhance the molecular permeation rate. Secondly, the pores in the inorganic particles themselves reduce mass transfer resistance while providing additional pathways for molecular transport. Among the various nanofillers, MOFs, as organic–inorganic hybrid materials, are regarded as promising candidates for modifying traditional polymer membranes. This is attributed to their high porosity, low thermal conductivity, and excellent compatibility with polymeric materials.

By taking advantage of the molecular sieving effect of MOFs, the resultant MMM is capable of circumventing the trade-off effect of traditional polymer membranes. Compared with conventional inorganic fillers such as zeolites, MOF-based MMMs exhibit a superior separation performance, as demonstrated in several recent excellent review articles [40,41]. However, the compatibility between MOF fillers and polymers is poor. The “trade-off” between the loading of the metal–organic framework material and the membrane integrity becomes more pronounced when the MOF loading is high. An increase in the loading amount of MOFs leads to particle agglomeration, while interfacial defects cause membrane rupture and permeation issues. In contrast, too low a load leads to an insufficient performance for membrane filtration efficiency or flux. Therefore, reducing the non-selective binding of hydrophobic polymers to MOF ligands and suppressing membrane wetting during long-term mesoporous dynamics are crucial.

(1) Selection and chemical modification of polymer matrix. Hydrophobic interactions and hydrophilicity have also been used to enhance the compatibility between the MOF filler and polymer [42]. Formation of covalent bonds between MOF fillers and polymer matrices is a more efficient strategy to generate defect-free MOF/polymer interfaces [43].
(2) MOF surface hydrophobic modification [44,45]. The surface functionalization of MOF fillers can enhance the bonding at the MOF/polymer interface [46]. The formation of non-covalent interactions and covalent bonds between MOFs and polymers can also enhance interfacial compatibility in MMMs [47]. This effectively alleviates the phenomenon wherein non-selective pores gradually become wetted during membrane operation due to excessive MOF loading.
(3) Introduction of interface layers between MOF and polymer matrix. The interface layer can bind metal ions and create nucleation sites for the growth of MOFs [48]. Interphase voids can be eliminated by using covalent or non-covalent bonding at the interface [49,50] and by introducing interfacial compatibilizers [51].
(4) Advanced methods for the preparation of MMMs. Currently, researchers have developed advanced methods for preparing MMMs, such as the gel–vapor deposition route [52] and interfacial polymerization [53]. By dynamically controlling the loading and film formation quality through various film preparation methods, the problem of film surface cracking under high loading conditions can be effectively resolved.

In summary, by appropriately selecting a polymer substrate and chemically modifying it to impart hydrophobicity to the MOF surface, creating an interfacial layer between the MOFs and the polymer substrate, and employing advanced preparation methods to fabricate the hybrid membrane, these approaches can effectively address the issue of reduced membrane integrity that occurs when high loads of MOFs are incorporated into the membrane. Table 2 summarizes the various metal–organic framework materials currently in use, their modification methods, and their applications in membrane distillation.

## 3. Research Status of MOF-Modified Membrane Distillation Membranes

**Figure 4:**
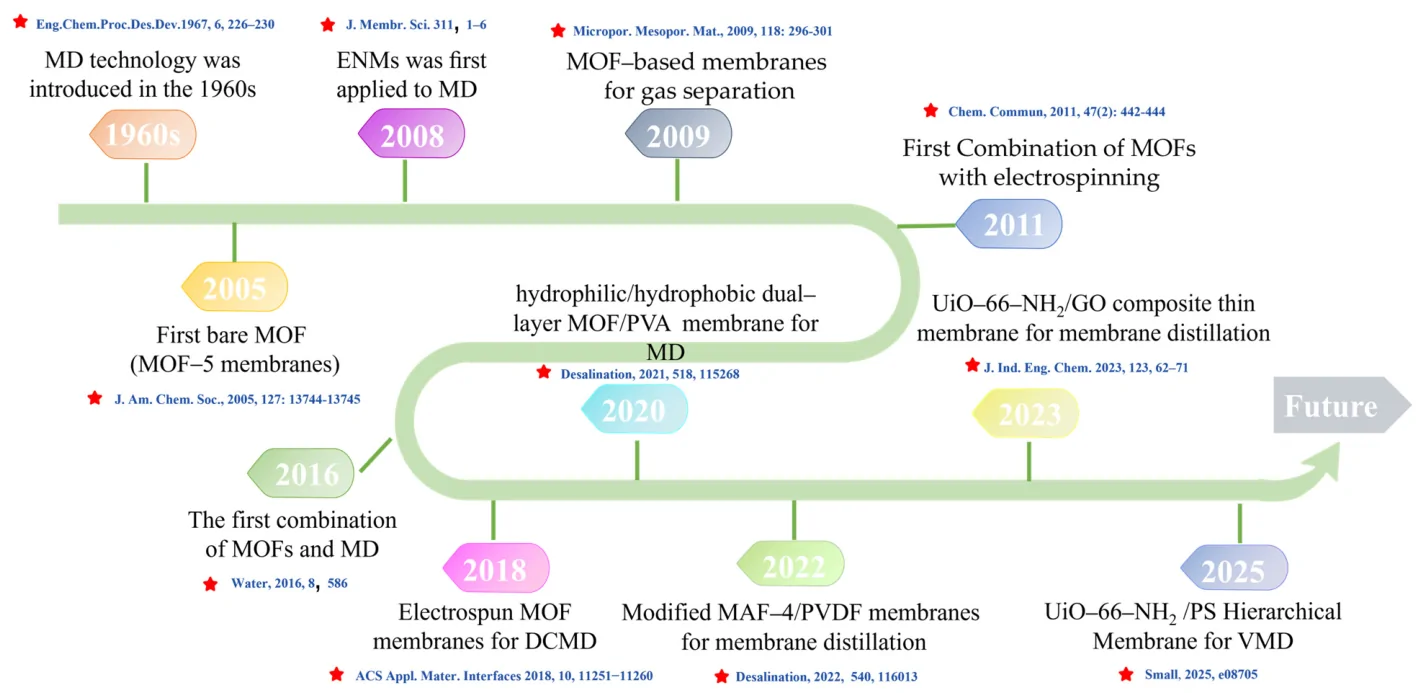
Illustrated history of membrane distillation and MOF development [33,63,64,66,67,71,75,77,78,79].

MD is a thermal, membrane-based separation process [73]. The driving forces for MD processes are quite different from other membrane processes, being the vapor pressure difference across the membrane rather than an applied absolute pressure difference and the concentration gradient or electrical potential gradient, which drives mass transfer through a membrane [73]. MD technology was introduced in the 1960s [74]. In 2009, MOF-based membranes were used for gas separation [75]. That same year, membrane distillation technology became commercially viable. Subsequently, various membranes suitable for membrane distillation were developed [76]. In 2015, MOFs and membrane distillation technology were combined for the first time [66]. Figure 4 summarizes the application potential and development history of MOFs in membrane distillation technology. In general, MOF-based membrane design strategies can be divided into two main categories. This section will focus on: (1) MOF polycrystalline membranes; (2) MOF-based composite membranes.

### 3.1. MOF Polycrystalline Film

MOF polycrystalline film is a membrane structure in which MOF crystals that grow well together serve as the selective layer [80]. The permeation selectivity and overall properties of the membrane are determined by the nature of the building units, pore size, and porosity of the MOFs. Therefore, it exhibits an excellent performance in the field of molecular separation [81]. However, preparing dense and defect-free MOF polycrystalline films on porous substrates still presents many challenges. Low mechanical strength limits its stability during long-term operation. Over the past decade, researchers have made significant efforts in developing methods for preparing MOF polycrystalline films, thereby advancing the research in this field. Currently, MOF polycrystalline films are mainly prepared by the in situ growth, secondary growth, and layer-by-layer growth methods [79,82].

#### 3.1.1. In Situ Growth Method

In situ growth is an effective method for preparing MOF membranes. The core concept is to mix MOFs with the supporting substrate in a single-precursor solution. The key advantage of this preparation method is that MOFs can be grown in situ on the surface of the substrate. Liu et al. [75] were the first to report on the preparation of MOF-5 membranes on porous alumina substrates, laying the foundation for subsequent research in this area. Chuang et al. [66] co-grew MOF crystals on alumina tube substrates and modified the surface of the MOF membrane by introducing perfluorinated molecules. Research has found that the modified membrane material not only retains the high stability and high water permeability of the alumina substrate but its surface modification also endows it with excellent hydrophobic properties. This has opened up entirely new research directions for designing next-generation, high-performance membrane distillation membranes for seawater desalination.

In terms of material surface modification, Huang and his research team [83] used bio-inspired polydopamine to modify the substrate surface, successfully synthesizing a ZIF-8 membrane with varying degrees of crystallinity. Polydopamine modification significantly enhanced the interfacial binding between the substrate and ZIF, thereby promoting the nucleation and in situ growth of ZIF-8. The prepared ZIF membrane performed excellently in seawater desalination applications, with an ion rejection rate of up to 99.8%. In addition, Huang et al. [61] successfully prepared dense and pure-phase UiO-66-NH_2_ membranes on aminopropyl-propoxyethoxysilane (APTES)-modified macroporous Al_2_O_3_ tubes. As the feed temperature increased, the water flux rose from 1.5 kg·m^−2^h^−1^ to 12.1 kg·m^−2^h^−1^, while the retention rate remained at 99.7%. Rahman et al. [65] synthesized pure UiO-66 crystals in situ on alumina hollow-fiber membranes. After modification via fluorosilane grafting, the modified membranes achieved a water flux of 14.95 L·m^−2^h^−1^ and a NaCl rejection rate of 99.9% during direct contact membrane distillation. Li et al. [84] prepared a pure-phase UiO-66 polycrystalline membrane on alumina hollow fibers using in situ solvothermal synthesis. This membrane exhibited excellent polyvalent ion retention properties and good permeability. Liu et al. [85] prepared a continuous aluminum MOF-303 membrane on an α-Al_2_O_3_ substrate using in situ hydrothermal synthesis. This film exhibits excellent retention efficiency for divalent ions while maintaining high permeability.

The high porosity of MOF polycrystalline membranes prepared by in situ growth provides additional pathways for the transport of water vapor molecules. However, due to the slow membrane growth process and stringent synthesis conditions, this approach remains challenging for the large-scale production of large-area MOF polycrystalline membranes. At the same time, MOF polycrystalline membranes are prone to crack defects and insufficient mechanical strength when subjected to stress, making them unable to meet the requirements for long-term operational stability.

#### 3.1.2. Secondary Growth Method

The secondary growth method consists of two steps. In the first step, a layer of MOF nanocrystals is uniformly and densely deposited on the surface of the porous substrate in advance. In the second step, the seed-treated substrate is placed in the MOF precursor solution. Under solvothermal conditions, the seeds nucleate and grow preferentially, ultimately resulting in a continuous MOF polycrystalline membrane with no major defects. Compared to the in situ growth method, this approach allows for better control over the crystal orientation, resulting in a crack-free, dense, and continuous membrane layer. Jin et al. successfully developed a ZIF-300(Zn) membrane on an alumina substrate using the secondary growth method for the first time. This membrane exhibited a high and stable performance: its permeability was 39.2 L·m^−2^h^−1^bar^−1^, while its retention rate for CuSO_4_ was 99.21% [86]. The quality of the seed layer is crucial for the membrane structure, and the seeds must be firmly anchored to the support. Crosslinking agents can effectively enhance the interaction between MOF seeds and porous substrates. Polyethyleneimine (PEI) can form hydrogen bonds with two organic linker groups in the MOF seeds as well as with free functional groups on the substrate surface, such as hydroxyl groups [87].

The secondary growth method enables the production of MOF membranes with uniform thickness, good orientation, and significantly improved properties. However, its process is more complex than the in situ growth method. Typically, nanoscale MOF crystals are required to obtain a satisfactory seed layer. Using this method directly to produce high-quality, large-area membranes remains challenging. Currently, there are few applications of membranes prepared by this method in seawater desalination via membrane distillation.

#### 3.1.3. Layer-by-Layer Growth

The layer-by-layer growth method is also called liquid-phase epitaxial growth. It is a self-assembly technique. Ultra-thin, uniform, and continuous MOF membranes are constructed layer by layer. This is done through alternating adsorption and reactions on the substrate surface. This method precisely adjusts membrane growth parameters (such as the precursor concentration and number of growth cycles), thereby enabling precise control over the membrane thickness.

Shekhah et al. prepared the first MOF-based thin film containing HKUST-1(Cu) using a layer-by-layer deposition method [88]. The layer-by-layer growth method is considered one of the most promising approaches for the large-scale production of MOF polycrystalline membranes. While this technique offers notable advantages—including strong interfacial bonding, minimal defects, and scalability—it requires significant amounts of organic solvent during membrane fabrication, thereby raising production costs. Furthermore, it is hindered by a lengthy preparation process and a low yield per growth cycle.

### 3.2. MOF-Based Composite Membranes

MOF-based composite membranes are fabricated by incorporating MOF particles as porous fillers into a polymer matrix. By integrating the exceptional properties of MOFs with the mechanical robustness of polymers, this strategy has gained considerable attention in the field of membrane distillation. Studies have demonstrated that this approach can overcome the traditional trade-off between selectivity and permeability in polymer membranes while also improving thermal stability and mechanical strength. As the core component of membrane separation, the structure of hydrophobic microporous membranes directly determines their separation performance, while the membrane fabrication process has a significant impact on the membrane’s structure. Compared to phase inversion membranes, their tortuous and closed pores limit vapor permeation, resulting in low water flux and wasted thermal energy [89]. The pore structure formed by the interconnection of fibrous membranes allows for high water flux (30 L·m^−2^h^−1^ at 40 °C). However, the randomly stacked and fluffy fiber structure leads to pore deformation, thereby reducing the membrane’s lifespan [90]. Compared with flat-sheet membranes, hollow-fiber membranes have relatively large specific surface areas [91], but the main impediment of the hollow-fiber module is its typically low flux (generally 1–4 L m^−2^ h^−1^ at 40–60 °C) [92]. This section systematically reviews the progress of the current research on MOF-based composite membranes, with an emphasis on their preparation methods.

#### 3.2.1. Electrospinning

**Figure 5:**
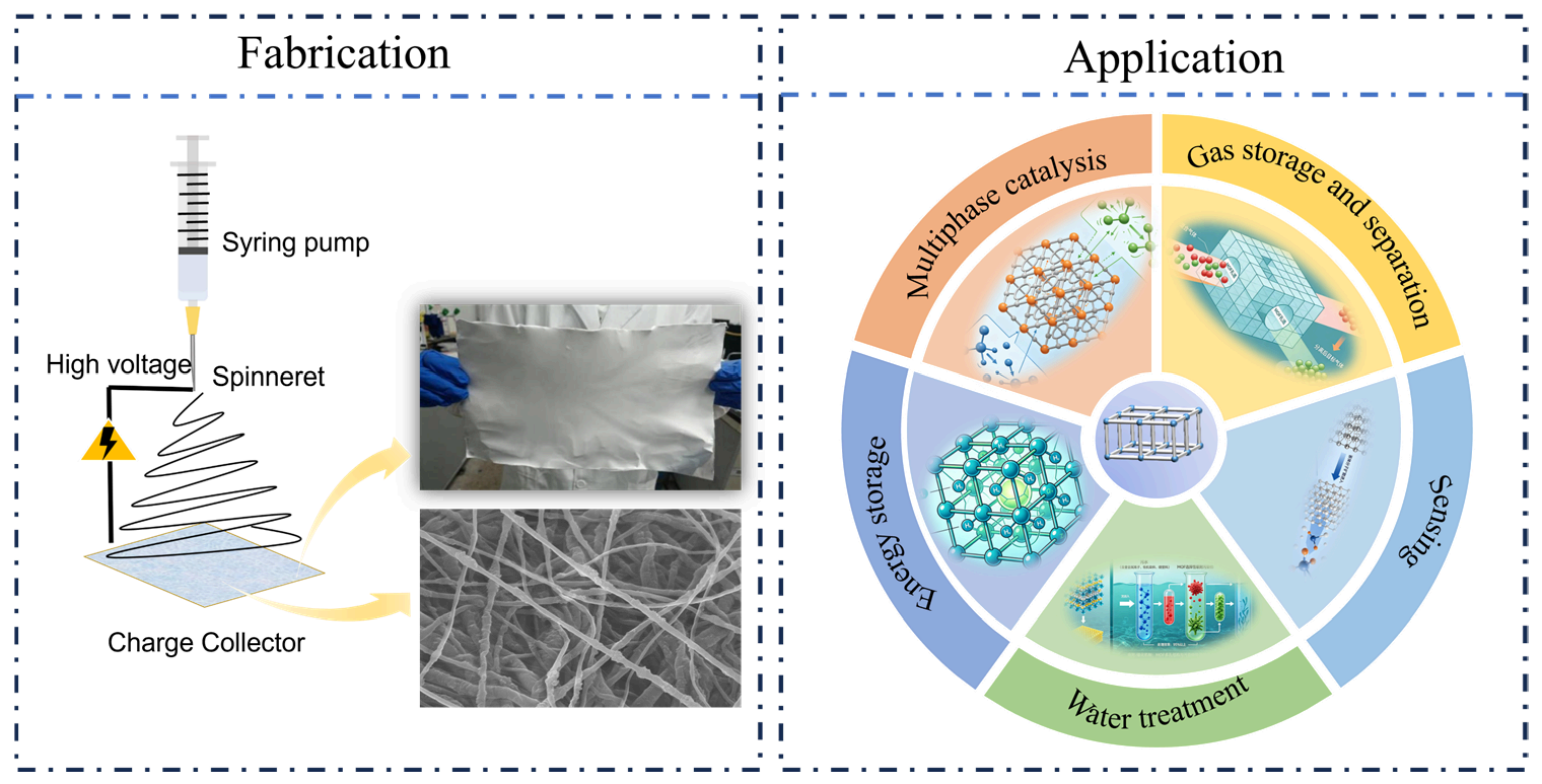
Preparation and applications of electrospun nanofiber membranes.

Electrospinning is an advanced technique for fabricating fiber membranes by stretching a charged polymer solution or melt in a high-voltage electric field to form nanofibers [88,93]. Figure 5 shows the preparation and applications of electrospun nanofiber membranes.

Since the first application of electrospun nanofiber membranes in the field of membrane distillation in 2008 [76], their high specific surface area, high porosity, adjustable fiber diameter and interconnected structure, made this technology the preferred choice for developing ideal materials for membrane distillation. MOF nanofiber membranes have a wide range of applications in gas separation and storage, catalysis, air pollutant filtration, and water treatment [94]. However, feed liquids in the water treatment field often contain surfactants and hydrophobic pollutants, leading to issues with membrane pore wetting and contamination in electrospun nanofiber membranes (ENMs). Therefore, researchers are focused on developing hybrid matrix membranes composed of organic–inorganic fillers and polymer nanofibers, thereby combining their advantages to improve the membrane’s physicochemical properties and separation performance. In particular, research on how MOFs, when combined with nanofibers as organic–inorganic fillers, endow the resulting MOF nanofiber membranes with excellent properties is ongoing. This section will systematically review the current research status of MOF nanofiber membranes in the field of membrane distillation. Table 3 also summarizes the relevant research findings.

In 2018, Yang et al. [67] prepared superhydrophobic polyvinylidene fluoride nanofiber membranes loaded with iron 1,3,5-benzenetricarboxylate MOFs at a concentration of up to 5 wt% on nonwoven substrates using electrospinning technology. The membrane remained stable during 5 h of operation. Compared to the original PVDF membrane (1.86 L·m^−2^h^−1^), the PV-5 membrane achieved a flux of 2.87 L·m^−2^h^−1^ at 48 °C. This represents a 54% increase in flux. Additionally, the NaCl rejection rate was as high as 99.99%. In 2020, Rana et al. [69] developed a three-layer nanofiber membrane structure. The top layer consisted of 5 wt% hydrophobic SiO_2_-PVDF nanofibers, the middle layer contained 1.5 wt% MOF-PAN nanofibers, and the bottom layer was made up of 1 wt% hydrophilic SiO_2_-PVDF nanofibers. The membrane flux increased with the increase in the amount of MOF added. A flux of 4.40 L·m^−2^h^−1^ was achieved during 5 h of direct membrane distillation operation, while an excellent retention performance was maintained. In 2021, Wu et al. [68] incorporated AlFu MOFs into polyvinylidene fluoride-co-hexafluoropropylene (PVDF-HFP) nanofiber membranes. The addition of AlFu MOFs had a positive effect on improving the membrane’s performance. Compared to the PVDF-HFP fiber membrane (10.6 L·m^−2^h^−1^), the nanofiber membrane with 2% AlFu MOFs added exhibited a flux of 22.8 L·m^−2^h^−1^, representing an 114% increase in performance. This demonstrates its excellent membrane distillation capabilities. In 2022, Huang et al. [58] incorporated ZIF-71 into PVDF-HFP to prepare hydrophobic electrospun nanofiber membranes. Compared to the PcH membrane’s flux of 14 L·m^−2^h^−1^, the ZIF-71/PcH membrane exhibited a significantly higher flux of 20 L·m^−2^h^−1^, representing an increase of 42.8%. In the same year, Wu et al. [33] prepared PVDF and MAF-4 composite membranes using electrospinning. Compared to the original PVDF nanofiber membrane (17.5 L·m^−2^h^−1^), the flux of the PVDF/MAF-4 composite membrane was 27.9 L·m^−2^h^−1^. The high-porosity MAF-4 provides additional steam transport pathways and excellent thermal insulation properties. In 2024, Jiang et al. [59] prepared core/shell-structured ZIF-CoZn@PVDF-HFP composite nanofiber membranes using coaxial electrospinning. The flux reached 21.8 L·m^−2^h^−1^, a 70% increase compared to the pure PVDF-HFP nanofiber membrane. In 2025, Yao et al. [64] prepared UiO-66-X/PS nanofiber membranes using electrospinning and hot-pressing techniques. The membrane achieved a flux of 137.6 L·m^−2^h^−1^, a desalination efficiency of 99.95% at 70 °C, and a vacuum pressure of −85 kPa, outperforming commercial PTFE (28.9 L·m^−2^h^−1^) as well as most reported polymer membranes.

The synergistic effect of the high porosity of the electrospun nanofiber membrane and the additional molecular transport pathways provided by MOFs significantly enhances the flux of MOF-based composite membranes. The key to improving the performance of fiber membrane distillation lies in the compatibility between MOFs and the polymer matrix, the structure of the MOFs themselves, and the amount of MOFs added.

#### 3.2.2. Coating Process Method

This process involves coating the surface of plate-shaped, cylindrical, or irregularly shaped components with a layer to create a thin film. The polymer concentration in the impregnation solution, impregnation time, and crosslinking agent concentration are the key process parameters of this method. These parameters affect the thickness of the coating material, the pore size of the membrane, and the integrity of the membrane structure [98]. Rahimpour et al. [55] coated a polyvinylidene fluoride substrate with an ultra-thin ZIF-8/chitosan composite layer, thereby enhancing the membrane distillation performance of the membrane for seawater desalination. Cao et al. [57] successfully synthesized nanoscale and micrometer-sized ZIF-8 particles by systematically studying the rules governing particle size control. After hydrophobic modification using POTS, these particles were deposited on a PTFE substrate via spraying. This membrane exhibits superior permeate flux compared to commercial PTFE membranes during the membrane distillation process. This research not only provides an innovative strategy for the preparation of superhydrophobic coatings but also expands the application potential of MOF materials as such coatings. Niu et al. [98] developed TFMOF/PDMS Janus membranes. Through the in situ growth of polyaniline, hydrophilic modification with polydimethylsiloxane (PDMS) was performed, followed by deposition of zirconium-based metal–organic framework (TFMOF) particles using a dip-coating process. This approach combines asymmetric wettability structures with photocatalytic membrane distillation to provide a solution for coal chemical wastewater treatment.

The coating process method is not only simple and easy to operate, with low equipment requirements and low costs, but it is also suitable for large-scale production. However, there are issues such as the poor interfacial compatibility between MOFs and the substrate, as well as declines in membrane permeability and mechanical properties under high loading conditions. Therefore, pretreating the substrate using plasma or surface grafting methods can significantly enhance the adhesion between it and the coating. By combining in situ growth techniques, directed loading of MOFs within the pores of the substrate can be achieved, thereby enhancing the practical application potential of this technology.

#### 3.2.3. Other Methods

The phase inversion method is based on the thermodynamically driven phase separation principle, converting a homogeneous polymer solution (or composite precursor solution) into a membrane material with a specific porous structure. First, the polymer is dissolved in a solvent to prepare a homogeneous polymer solution. Subsequently, a film with a thickness of 100–200 μm is prepared. Finally, the prepared polymer film is immersed in a coagulation bath using water as the non-solvent, causing the polymer to precipitate out of the solution. Rahimpour et al. [55] developed a new type of thin-film composite (TFC) membrane by coating an ultra-thin layer of ZIF-8/chitosan on the surface of a PVDF substrate. The film was treated by immersing it in a non-solvent bath using the phase transition method. The results showed that the ZIF-8/chitosan-modified membrane had a NaCl retention rate of over 99.5%. Xie et al. [70] successfully prepared a novel hydrophobic hybrid PVDF hollow-fiber membrane doped with AlFu MOFs using the dry spray–wet phase transition method. The AlFu MOF additive has a positive effect on improving the membrane performance.

Solution casting is a technique in which a solution is applied to a substrate, and then the solvent is evaporated to form a thin film. Xie et al. [71] prepared hydrophilic/hydrophobic bilayer membranes via solution casting on a hydrophobic microporous PTFE substrate. With the adjustment using nano-porous AlFu MOFs, the water vapor flux reached 45.1 L·m^−2^h^−1^, while a 99.9% retention rate was maintained in the direct contact membrane distillation (DCMD) process.

Vacuum filtration is a classic and efficient method for preparing metal–organic framework membranes. Using the vacuum negative pressure difference as a driving force, The suspension containing MOF nanoparticles (or precursors) is passed through a porous support, allowing the MOF particles to quickly accumulate and form a continuous, dense film layer on the surface or within the pores of the support material. This method is simple to operate, has a short preparation time, and allows for easy control of the membrane thickness. Chen et al. [62] modified UiO-66-NH_2_ using PVA as a crosslinking agent. The modified UiO-66-NH_2_ was then immobilized on a PTFE membrane through vacuum filtration, resulting in the development of a new type of Janus composite membrane. In the 24 h DCMD experiment, a desalination rate of 99.99% was achieved, with a flux of 21.3 L·m^−2^h^−1^. The research team also used a crosslinking agent to fix GO and UiO-66-NH_2_ onto the PTFE hydrophobic membrane. Compared to the original PTFE membrane, the Janus membrane exhibits superior moisture resistance. The flux was maintained at 21.2 L·m^−2^h^−1^, and the retention rate reached 99.9% after 48 h of continuous operation [66]. Moriones et al. [99] deposited MOF@GO nanohybrids onto two layers of polydopamine (PDA) positioned on top of a commercial polyethylene (PE) film using vacuum filtration. Compared to the GO membrane (5 L·m^−2^h^−1^), the MOF@GO membrane has a higher flux (13 L·m^−2^h^−1^).

In summary, the addition of MOFs not only increases membrane flux but also enhances the membrane’s water-repellent properties, demonstrating significant potential applications in the field of membrane distillation for seawater desalination. MOF polycrystalline films possess desirable properties due to their customizable pore structures, excellent hydrophobic/superhydrophobic control capabilities, high chemical stability, and good thermal stability, providing a new material option to address the bottleneck issues in traditional membrane distillation processes, such as membrane fouling, low permeate flux, and low thermal efficiency. MOF composite membranes have significant advantages over MOF polycrystalline membranes in terms of scalability and cost control, demonstrating strong commercial potential.

## 4. The Working Principle of MOFs in Membrane Distillation

**Figure 6:**
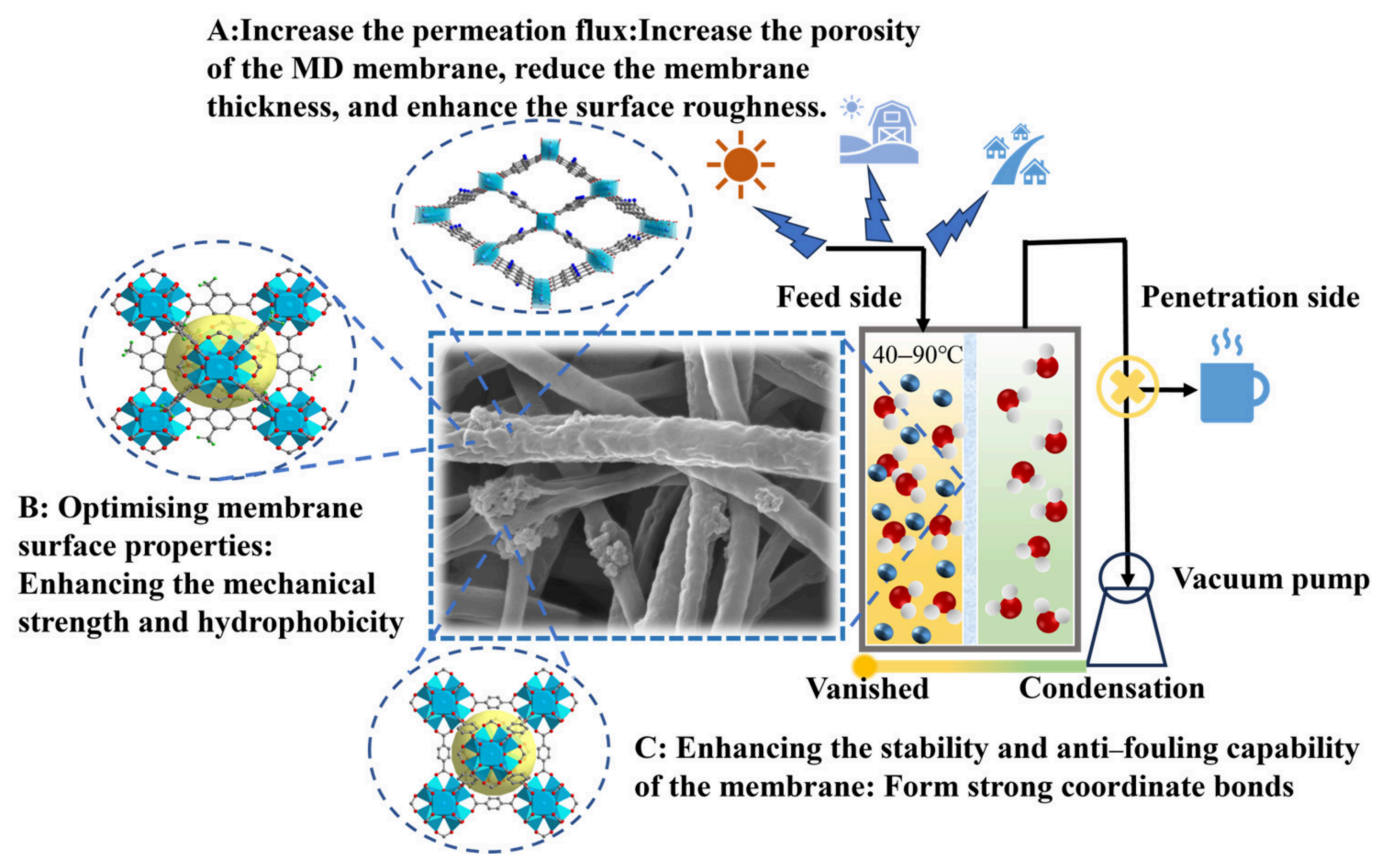
Schematic diagram of working principle of metal–organic frameworks in membrane distillation.

Metal–organic frameworks possess high porosity, extremely large specific surface areas, and customizable pore structures. MOFs regulate the pore structure, hydrophobicity, thermal stability, and anti-fouling properties of the membrane. Itroducing them into the membrane distillation system for the preparation of MOF-based membranes can significantly enhance the flux and long-term operational stability of membrane distillation. This section explores the working principles of MOFs in membrane distillation. The key mechanisms include enhancing permeate flux, optimizing membrane surface properties, and improving membrane stability and anti-fouling capabilities, as illustrated in Figure 6.

### 4.1. Increasing Permeate Flux

MOFs enhance permeation flux by increasing the porosity of MD membranes, reducing membrane thickness, and increasing surface roughness [56]. Increasing the porosity of the MD membrane enhances the effective surface area available for water vapor evaporation, thereby providing more pathways for transport [100]. Cheng et al. [70] prepared hollow-fiber hydrophobic membranes doped with AlFu MOFs. The AlFu MOF material converts the originally dense PVDF membrane structure into porous, while enhancing the mass transfer pathways of the membrane. The increase in effective porosity not only enhances the membrane’s pore size but also reduces the tortuosity of the membrane pores [101]; reducing the thickness of the MD membrane shortens the actual distance that water vapor must travel through the membrane, thereby helping to decrease the mass transfer resistance. Guo et al. [102] incorporated copper-based metal–organic framework (Cu-CAT) materials into hydrophilic/hydrophobic polyvinylidene fluoride nanofiber membranes, creating photothermal-responsive Janus nanofiber membranes. This approach reduced the thickness of the photothermal layer by 15 μm. Increasing the surface roughness of MOFs creates interfacial gaps, thereby providing more pathways. Additionally, the greater the temperature polarization coefficient, the more severe the effective transmembrane temperature difference loss, resulting in lower flux. MOFs inherently have better thermal conductivity than pure hydrophobic polymers, effectively reducing the temperature polarization coefficient and thereby enhancing the driving force for steam mass transfer. The permeate flux of the membrane is proportional to its porosity and inversely proportional to its thickness and curvature [103]. Too large an aperture will affect the solute retention efficiency, while too thin a membrane thickness will impair its mechanical properties. Therefore, an ideal MD membrane needs to achieve a balance among the retention rate, mechanical strength, and permeate flux in order to fulfill its potential for practical applications.

### 4.2. Optimizing Membrane Surface Properties

Composites formed by MOFs and a matrix can enhance the interactions between molecular chains in the membrane material, thereby improving the membrane’s mechanical strength and toughness. This allows the membrane to withstand external forces in harsh water environments while reducing the risk of membrane rupture [104]. In addition, the surface chemical properties of MOFs can be manipulated through ligand modification. Introducing fluorine- or alkyl-containing organic ligands into the MOF structure, or performing hydrophobic modifications on the MOFs, can significantly enhance the surface hydrophobicity of the membrane. Chuang et al. [66] synthesized MOF crystals in symbiosis on alumina tube substrates and then introduced perfluorinated molecules onto the surface of the MOF-functionalized membrane. This membrane retains the high water permeability and resistance to high temperatures and pressures inherent in the alumina substrate while also possessing a hydrophobic surface. Xu et al. [45] developed a novel porous, hydrophobic MOF-801@GO-PDMS membrane. PDMS contributes to the membrane’s hydrophobic stability by utilizing its inherent hydrophobicity and the crosslinking of its molecular fragments.

### 4.3. Improving Membrane Stability and Anti-Fouling Properties

As the operating time of membrane distillation is prolonged, solutes gradually accumulate on the membrane surface and clog the pores, resulting in reduced flux and membrane wetting, ultimately leading to damage to the membrane structure [105]. By optimizing the metal/ligand composition and surface modification of MOFs, the stability of the membrane under extreme pH conditions and its tolerance in extreme temperature environments can be enhanced [39,106]. Highly charged metal ions with high charge densities form strong coordination bonds with oxygen-containing ligands such as carboxylate groups, effectively resisting acidolysis caused by attacks from hydrogen ions (H^+^) or substitution by hydroxyl groups (-OH). The UIO series, which contains high-valent metals such as Zr^4+^ and Hf^4+^, exhibits strong acid resistance and can operate effectively for extended periods in high-salinity and highly acidic environments. It still maintains good structural integrity and acid resistance, making it a candidate material for numerous research projects currently. In contrast, MOFs formed from low-cost metals have weaker bonding energies and tend to undergo hydrolysis in non-neutral pH environments, leading to the collapse of their crystal structures. In general, by selecting appropriate metals/ligands and modifying the surface, the stability of MOFs under extreme pH conditions can be effectively improved.

### 4.4. The Influence of Other Factors on the Membrane Evaporation Performance

Other factors that affect the performance of membrane distillation include the temperature, feed flow rate, and feed composition. As the temperature rises, the viscosity of the liquid decreases, the thermal motion of water molecules accelerates, the molecular diffusion coefficient increases, and the driving force for mass transfer across the membrane becomes stronger. As a result, the water flux continues to increase as the temperature rises. The fluidity of the feed solution is high, allowing it to quickly wash away the concentration polarization layer on the membrane surface. This reduces the accumulation and adhesion of salts on the membrane surface, thereby significantly increasing the water flux. With a single-feed liquid, the larger the ion particle size, the better the retention effect. When the feed contains impurities, a contaminant layer forms on the membrane surface, blocking the permeation channels and reducing the salt retention rate. At high salt concentrations, the osmotic pressure of the solution increases dramatically. Water molecules have difficulty penetrating the solution, and the concentration gradient of salt ions becomes too large, resulting in a significant decrease in the efficiency with which salts are retained.

## 5. Development Trends and Challenges of MOF-Modified Membranes

### 5.1. Improving the Hydrophobicity of MOF Membranes

**Figure 7:**
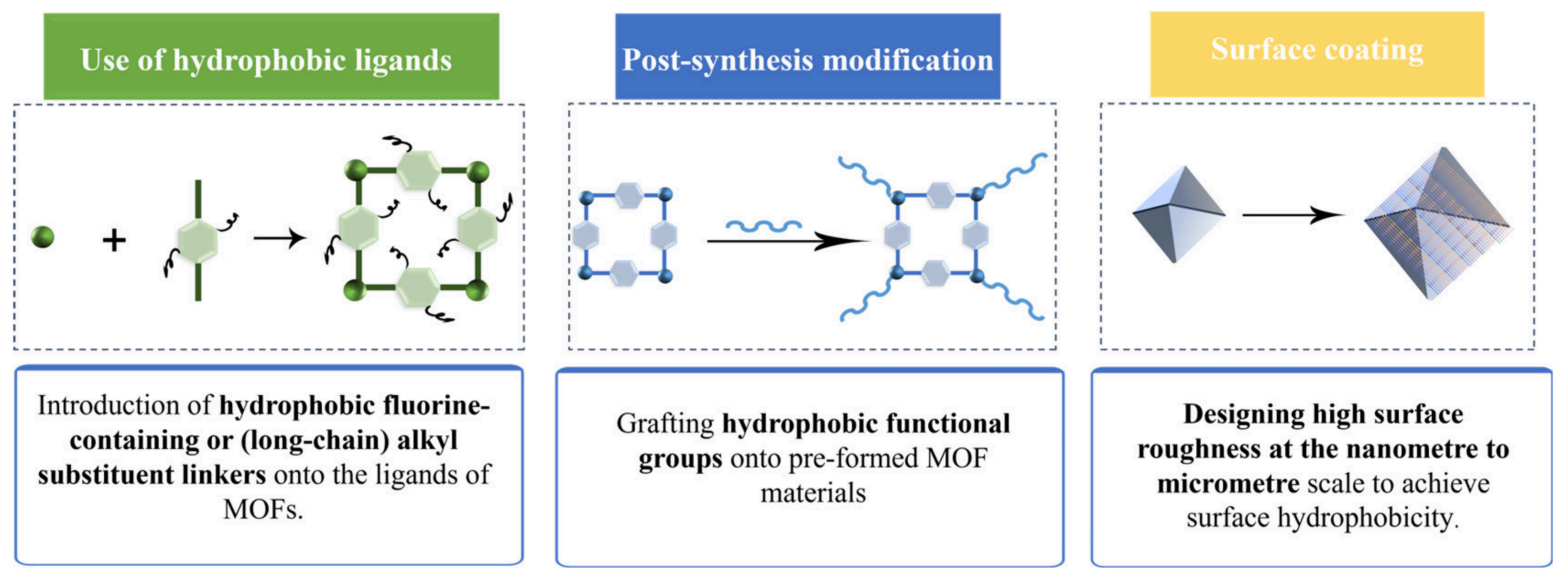
Strategies for fabricating hydrophobic MOFs and composites.

Membrane distillation requires superhydrophobic or fully hydrophobic membranes to prevent pore wetting. Pore wetting occurs when the aqueous phase penetrates the membrane, resulting in the loss of the membrane’s hydrophobic properties [107]. Inspired by hydrophobic natural surfaces such as lotus leaves, gecko feet, and mosquito eyes, highly hydrophobic membranes can be designed and fabricated to improve wetting resistance. As shown in Figure 7, there are three strategies for synthesizing hydrophobic metal–organic framework materials: (i) synthesis using organic linkers decorated with alkyl or fluorinated groups; (ii) the post-synthetic modification of MOFs with hydrophobic groups; (iii) the introduction of external surface corrugation using aromatic hydrocarbon building blocks; and preparation of hierarchical porous hydrophobic MOF composites whose overall hydrophobicity may be due to the use of an intrinsically hydrophobic MOF or a hydrophobic two-dimensional material [108]. Shi et al. [44] synthesized superhydrophobic and superoleophilic MOF materials via an amide reaction between amino groups and octadecyloyl chloride. The introduction of long alkyl chains reduced the surface free energy, enabling the successful preparation of a superhydrophobic zirconium-based metal–organic framework (UiO-66-NH-C18). Liu et al. [109] proposed that thermal activation can remove hydroxyl groups from the framework of MOFs, thereby creating a hydrophobic surface that improves the membrane’s resistance to wetting. The resulting hydrophobic MOF membrane exhibited a water flux of 12.8 L·m^−2^·h^−1^ and a NaCl rejection rate exceeding 99.9%. Therefore, the preparation of hydrophobic MOF-based membranes is one of the directions for future development.

### 5.2. Achieving Large-Scale Production of MOF Membranes

The key to the large-scale production of MOF membranes lies in developing a process that allows for continuous manufacturing. Wang et al. [110] reported a thermally induced phase separation hot-pressing (TIPS-HoP) strategy. Ten different MOF membranes can be prepared using the roll-to-roll method. While this technology provides an approach for preparing defect-free MOF membranes, the large-area, continuous production of such membranes remains a significant challenge [80]. However, the industrial production of MOF membranes has not yet been achieved, mainly for the following three reasons: First, in the laboratory, small-sized carriers are commonly used for preparing MOF membranes, allowing for the production of samples with uniform properties. However, in large-scale production, large-sized, high-throughput carriers are required, making it difficult to maintain uniformity and stability. Second, the high synthesis costs of MOF materials themselves results in elevated production costs for MOF membranes. Third, a process for the large-scale production of MOF membranes has not yet been developed. The laboratory preparation method is too complicated to enable continuous operation.

### 5.3. Improving the Operational Stability of MOF Membranes

Most MOFs (such as ZIF-8, UiO-66) are prone to crystal dissolution, structural collapse, or ligand loss under conditions of high humidity, highly polar solvents, or extreme pH values [111]. MOFs with weak metal–ligand bonds are susceptible to attack by water molecules, limiting their application in seawater desalination [112,113]. Therefore, the design and development of hydrophobic MOFs are of great significance for the application of MOFs in aquatic environments [44,102]. Methods for synthesizing hydrophobic MOFs include the one-step and post-synthesis modification methods [114]. The one-step method involves using ligands with hydrophobic units to construct hydrophobic MOFs. The post-synthesis modification method involves the hydrophobic grafting or coating of the prepared MOFs. To realize many important water-related applications, MOF materials should exhibit both thermodynamic and kinetic hydrolytic stability. Thermodynamic stability here refers to strong coordination bonds between the inorganic node and organic linker that allow for enhanced chemical stability in aqueous and other media under normal and harsh conditions. Kinetic hydrolytic stability, in the context discussed here, is related to the steric effect for preventing the intrusion of water molecules into the framework and subsequent hydrolysis reaction, which breaks the metal–ligand bonds [115]. There are two methods to improve the stability of MOFs. One involves using high-valent metal cations (such as trivalent metals like Al^3+^, Fe^3+^, and Cr^3+^ and tetravalent metals like Zr^4+^, Hf^4+^, and Ti^4+^) as hard acids, along with carboxylate ligands, to create stable frameworks [116]. With the same connecting groups and coordination environment, higher-valent metal cations can form stronger and more cohesive coordination bonds. This leads to the formation of a more rigid framework structure and enhances the hydrothermal stability of the MOF. A typical example of such structures is the MIL series (where “MIL” stands for Institut Lavoisier materials). The second method involves using softer azole linker groups (such as the imidazolate, triazolethane, tetrazolylthio, and pyrazolylthio groups) as ligands. The nitrogen-containing azole ligands can form more stable interactions with softer divalent metals and other low-valent metal cations, thereby enabling the formation of robust MOF structures. A typical example of such structures is ZIFs.

## 6. Summary

The ability of MOFs to have regularly structured pores and surface sites that can be easily functionalized makes them excellent candidates for membrane distillation. Although some fundamental research achievements have been made in the field of membrane distillation using MOF polycrystalline membranes and MOF-based composite membranes, industrial application has not yet been achieved. The main challenges include the following three aspects:

(1) Scalable film-making equipment has not yet been developed, making it impossible to meet the demands of large-scale industrial production.
(2) The cost issue associated with the processing and synthesis of MOF materials remains a significant challenge.
(3) Fundamental issues such as the particle agglomeration, poor compatibility, easy degradation, and limited loading capacity of MOFs during film formation require further research.

With the continuous development of interdisciplinary research and technological advancements, MOF membranes are expected to become key technologies in areas such as high-salinity wastewater treatment and seawater desalination. They will provide important technical support for addressing global water shortages and achieving the “dual carbon” goals.

## Figures and Tables

**Table 1:** Different types of membranes used in membrane distillation.

Membrane Type	Common Materials	Advantages	Insufficiencies	Ref.
Inorganic Membrane	Ceramics (Al_2_O_3_, ZrO_2_, TiO_2_), metals (stainless steel, titanium alloy), glass	High-temperature-resistant; acid and alkali corrosion-resistant; high mechanical strength; good chemical stability; uniform pore size distribution; strong resistance to contamination; long service life.	High preparation costs; brittle membrane that breaks easily; difficult processing; high costs for large-scale application; complex membrane module design.	[17,18]
Polymer Membrane	Polyvinylidene fluoride (PVDF), Polytetrafluoroethylene (PTFE), Polypropylene (PP), Polysulfone (PSF)	Simple preparation process and low cost; good flexibility and easy to shape; suitable for mass production; flexible membrane module design.	Poor high-temperature resistance (usual operating temperature <100 °C over long periods); prone to swelling in organic solvents; weak resistance to contamination; rapid aging and flux degradation with prolonged use.	[19,20,21]
Mixed Matrix Membrane	Polymer substrates (PVDF, PTFE, etc.) + inorganic fillers (graphene, MOFs, ceramic particles, carbon nanotubes, etc.)	Combines the flexibility and processability of polymer membranes with the advantages of inorganic fillers, such as high-temperature resistance, pollution resistance, and high porosity. Results in better flux and retention rates compared to single-polymer membranes.	Inorganic fillers have poor compatibility with polymer substrates, tend to agglomerate, require complex preparation processes, are difficult to disperse within the polymer, and result in higher costs compared to pure polymer films.	[19,22,23]

**Table 2:** Metal–organic frameworks for membrane distillation and their modification methods.

MOF Type	MOF Name	Modification Method	Application Scenario	Ref
ZIF Series	ZIF-8	Modified metal oxide doping	Membrane distillation	[54]
	ZIF-8	Doping	Membrane distillation	[55]
	ZIF-8	CNT functionalization	Removing antibiotics from wastewater from the pharmaceutical industry	[56]
	ZIF-8	POTS modification	Membrane distillation	[57]
	ZIF-71	Doping	Dye wastewater treatment	[58]
	ZIF-8	Doping	Anti-pollution membrane distillation	[33]
	ZIF-8	Doping	Membrane distillation	[59]
UiO Series	UiO-66\UiO-66-NH_2_	Doping	Research on desalination performance	[60]
	UiO-66-NH_2_	APTES-modified a-Al_2_O_3_ tube	Seawater desalination	[61]
	UiO-66-NH_2_	Polyvinyl alcohol (PVA) modified with a crosslinking agent	fouling and wettability in membrane distillation	[62]
	UiO-66-NH_2_	GO	Membrane distillation	[63]
	UiO-66-NH_2_	Doping	Membrane distillation	[64]
	UiO-66	Silane-surface-modified impregnation grafting technique	Membrane distillation	[65]
MIL Series	NH_2_-MIL-53(Al)	MOF-functionalized alumina tube	Membrane distillation	[66]
Other types of MOFs	Fe-BTC	Electrospinning solution doping	Membrane distillation	[67]
	AlFu MOF	Electrospinning solution doping	Membrane distillation	[68]
	MOF-808	Electrospinning solution doping	Membrane distillation	[69]
	AlFu MOF	Doping	Membrane distillation	[70]
	AlFu MOF	Doping	Membrane distillation	[71]
	UiO-66/MIL-101(Cr)/ZIF-8	Doping	Water treatment	[72]

**Table 3:** Electrospun MOF nanofiber membranes for membrane distillation.

MOF Membrane	MOF Type	MD Configuration	Feed	Temperature (°C)	WCA (°)	Flux (L·m^−2^h^−1^)	Retention Rate (%)	Ref.
MOF-F300/PVDF	MOF-F300	DCMD	3.5% NaCl	48	138.06 ± 2.18	2.87	99.9	[67]
PAN-MOF	MOF-808	DCMD	35 g L^−1^	46 ± 1.5 °C	140.8	4.4	99.9	[69]
P/AlFu-2	AlFu MOF	DCMD	3.5% NaCl	40	135 ± 0.3	22.8	99.9	[68]
ZIF/PcH	ZIF-71	DCMD	35 g L^−1^	60	134 ± 1.2	20	99.9	[58]
PVDF/MAF-4	MAF-4	DCMD	35 g L^−1^	60	/	27.9	99.9	[33]
ZIF-CoZn@PVDF-HFP	ZIF-CoZn	DCMD	35 g L^−1^	60	144	21.8	/	[59]
PVDF-HFP/UiO-66-NH_2_	UiO-66-NH_2_	DCMD	100 mg/L ammonia solution (pH = 11)	60	/	/	/	[95]
ZIF-71/PH-PSF	ZIF-71	DCMD	35 g L^−1^	60	147.6°	/	/	[96]
ZIF-8/PVDF-HFP	ZIF-8	DCMD	/	40	/	44.5	99	[97]
UiO-66-NH_2_/PS	UiO-66-NH_2_	VMD	3.5% NaCl	70	/	137.6	99.9	[64]

## Data Availability

No new data were created or analyzed in this study. Data sharing is not applicable to this article.

## Abbreviations

MD	Membrane distillation
MOFs	Metal–organic frameworks
RO	Reverse osmosis
FO	Forward Osmosis
BTC	Benzene-1,3,5-tricarboxylic acid
BDC	Bind terephthalic acid
PVDF	Polyvinylidene fluoride
PTFE	Polytetrafluoroethylene
PP	Polypropylene
PSF	Polysulfone
PE	Polyethylene
PAN	Polyacrylonitrile
PVA	Polyvinyl alcohol
PVDF-HFP	Polyvinylidene fluoride-co-hexafluoropropylene
PDA	Polydopamine
TIPS-HoP	Thermally induced phase separation hot pressing
TA	Tannic acid
MMMs	Mixed matrix membranes
TFN	Thin-film nanocomposite
TFC	Thin-film composite
POTS	1H,1H,2H,2H-perfluorooctyltriethoxysilane
PDMS	Polydimethylsiloxane
GO	Graphene oxide
ENMs	Electrospun nanofiber membranes
ZIF-8	Zeolite imidazolate framework
AlFu MOF	Aluminum fumarate metal–organic framework
DCMD	Direct contact membrane distillation
